# Biosynthesis of reduced graphene oxide using *Turbinaria ornata* and its cytotoxic effect on MCF‐7 cells

**DOI:** 10.1049/nbt2.12057

**Published:** 2021-05-18

**Authors:** K. M. Smita, L. Stanley Abraham, V. Ganesh Kumar, Raguraman Vasantharaja, R. Thirugnanasambandam, Ajit Antony, K. Govindaraju, T. Senthil Velan

**Affiliations:** ^1^ Centre for Ocean Research Sathyabama Institute of Science and Technology Jeppiaar Nagar, Rajiv Gandhi Salai Chennai India

## Abstract

Graphene‐based nanomaterials are gaining importance in biomedicine because of their large surface areas, solubility, and biocompatibility. Green synthesis is the most economical method for application, as it is rapid and sustainable. Biofunctionalized reduced graphene oxide (TrGO) nanosheets were synthesized using methanol extract of *Turbinaria ornata*, and bioreduction of graphene oxide was primarily confirmed and characterized using UV‐visible, Fourier transform infrared (FTIR), and X‐ray diffraction spectroscopy and further characterized by zeta potential and transmission electron microscopy. The FTIR spectra of TrGO showed a decrease in the band intensities of oxygen groups, thus confirming effective deoxygenation. The zeta potential value of −34.6 mV revealed that synthesized TrGO was highly stable. The cytotoxic effect of TrGO against MCF‐10A and MCF‐7 cells was ascertained using MTT assay, showed a greater cytotoxic effect on MCF‐7 cells. The IC_50_ of TrGO treatment against MCF‐7 was calculated to be 31.25 µg, which is onefold lower than the cytotoxic effect of methanolic extract of *T. ornata* (60.0 ± 1.14 µg/ml). In addition, there was a statistically significant difference in cell viability between MCF‐10A and MCF‐7 cells in the treatment of TrGO. Hence, this study results in an efficient green reductant for producing rGO nanosheets that possess cytotoxicity against breast cancer cells.

## 1 INTRODUCTION

Graphene has been a fascinating nanomaterial in the therapeutics field with various biomedical applications such as drug delivery, gene therapy, and antibacterial and anticancer agents [1]. Due to its thin layered sheet structure and large surface area, it has exceptional electronic, mechanical, and biological properties [2]. Graphene nanoparticles are biocompatible, can achieve targeted drug delivery by acting as a drug, and have many potential applications in various devices and composites [3, 4] because of their unique physical and chemical properties [5, 6].

Precisely, oxidized graphene oxide (GO) and reduced graphene oxide (rGO) are considered potential agents for drug delivery and therapeutic application based on their biocompatibility [7]. In general, Hummers' method is used to synthesize GO and involves oxidative exfoliation of graphite using H_2_SO_4_/KMnO_4_ [8]. Although chemical methods of reducing GO are advantageous, the formation of irreversible aggregates due to strong van der Waals binding forces around the graphene planes leads to bottlenecks and constrains its successful processing [9, 10, 11]. To overcome such limitations, an alternative approach that specifically involves the application of green materials such as plant extracts, bacteria, and biomolecules as ‘eco‐friendly’ agents for synthesizing reduced GO is receiving greater attention [12, 13]. The extracts of different biological materials act as ‘green reductants’ for the effective synthesis of GO for application in biomedicine. Among the plant extracts, marine seaweeds, especially red and brown macroalgae, have proven to be efficient bioreducing agents [14]. Aqueous extract of the red macroalgae *Kappaphycus alvarezii* (Phyco‐sap) containing flavonols and transition metals was documented for partial reduction of GO for application as liquid fertilizer [15]. Solid granules of the brown macroalgae *Sargassum tenerrimum*‐functionalized GO composites (Fe_3_O_4_/Fe, SnO_2_/SnO/Sn, or ZnO/Zn‐functionalized GNs) were found to be non‐toxic to human lung carcinoma cells (A549), but the study has reported that biosynthesized graphene nanosheets (GNs) with high surface areas ensured a green, sustainable, and cost‐effective approach for efficient removal of excess fluoride from fluoride‐rich drinking water [16]. A study on phaeophycean macroalgal biomass‐derived bio‐oil from *Macrocystis pyrifera* for the hydrothermal process of synthesizing green‐emitting graphene oxide‐carbon dot (GO‐CD) composites has reported an increase in seed germination of mung bean [17].

*Turbinaria ornata* (Phaeophyceae) is an important marine brown algae commonly available in coral reef crests. Seaweeds are proven to be abundant sources of polysaccharides and several bioactive compounds such as fucoids and sulphated polysaccharides [18]. These brown algae possess broad‐spectrum biological properties including antibacterial [19], anticoagulant [20], anti‐inflammatory; and anti‐oxidant properties [21]. Most importantly, bioactive sulphated polysaccharide‐fucoidan derived from *T. ornata* has immense pharmaceutical properties such as anticancer [22]; antiviral; myocardial injury prevention [23]; hepatoprotective [24]; and neuroprotective activities [25]. Brown algae are well exploited for their medicinal value [26, 27], and their extracellular polysaccharides are used for the biosynthesis of AuNPs [28]. A recent study has documented the fungicidal effect of a methanolic extract of *T. ornata* against *Candida albicans* [29].

The present study discusses the seaweed extract‐based reduction of GO using methanolic extract of brown macroalgae *T. ornata*; analyzing the spectroscopic and morphological properties using spectroscopic instruments like UV‐visible, Fourier transform infrared (FTIR), X‐ray diffraction (XRD), DLS, EDX, and transmission electron microscopy (TEM) and elucidating its biological efficacies through *in‐vitro* anticancer by MTT against human breast carcinoma (MCF‐7) and against normal human breast epithelial cell lines (MCF‐10A).

## 2 MATERIALS AND METHODS

### 2.1 Materials and chemicals

Brown seaweed *T. ornata* was collected from the Rameswaram‐Mandapam, Ramnad District, and washed thoroughly with tap water to remove epiphytic growth and surface adhering salts. Then shade dried and pulverized. The pulverized seaweed was sieved with a 0.5 mm mesh and kept in a polyethylene bag at 4°C until use.

Methanol, graphite flakes, Sulphuric acid (H_2_SO_4_), nitric acid (HNO_3_), potassium permanganate (KMnO_4_), hydrogen peroxide solution (H_2_O_2_) were purchased from Merck (Mumbai). DMSO and MTT reagent were purchased from SRL Chemicals, India. RPMI‐1640 medium, Foetal bovine serum (FBS), penicillin, and streptomycin were purchased from Himedia India Pvt Ltd.

Cell lines were procured from NCCS, Pune, India, and the cytotoxicity assay was carried out at Lifeteck Research Centre, Chennai, India.

### 2.2 Methanolic extraction of *Turbinaria ornata*

The seaweed extract was carried out by dissolving 10 g dried biomass of *T. ornata* in 100 ml methanol (AR grade) and maintaining a stirring condition using an orbital shaker for 24 h at room temperature (28°C). The solution was then filtered using Whatman filter paper No. 42 and dried in a water bath. The final extract was reconstituted with 100 ml of deionized water and used for the experimental analysis.

### 2.3 Preparation of graphene oxide

Synthesis of GO was carried out by modified Hummers' method [30]. In brief, 2 g of graphite flakes were treated with 80 ml H_2_SO_4_ and later with 20 ml of HNO_3_, and using a magnetic stirrer, the solution was stirred for 30 min in an ice bath. KMnO_4_ (12 g) was slowly added to the mixture and stirred continuously for 10 min. The reaction mixer was heated in a water‐bath at 35°C for 30 min. The reaction mixture was then diluted with 160 ml of deionized water. After 1 h, the reaction mixture was further diluted with the addition of 400 ml of deionized water, and then 12 ml of 30% v/v hydrogen peroxide solution was slowly added. Following these steps, the black‐coloured graphite solution became brownish‐yellow. Separation of precipitated graphite oxide was centrifuged at 3000 rpm/min for 15 min. Later, the precipitate was washed and then resuspended in deionized water followed by sonication for 3 h to facilitate the exfoliation of stacked graphite oxide sheets into monolayer or multilayered GO sheets. The above prepared GO brown solution (5 mg/ml) was used for further experiments. Then the GO solution was filtered and vacuum dried for further characterization.

### 2.4 Synthesis of reduced graphene oxide nanosheets [15]

The green method of reduction was carried out by dispersing GO (5 mg/ml) in 2 ml of deionized water and sonicated for 30 min, followed by 8 ml methanol extract of *T. ornata* at room temperature. The subsequent reaction solution was heated at 60°C in a water bath until the colour of the suspension turns to black. Thus obtained, rGO was isolated by centrifugation at 3000 rpm/min for 15 min. The black pellet was washed repeatedly with deionized water and vacuum dried for further characterization.

### 2.5 Spectroscopic and morphological characterization

UV‐visible spectra of the aqueous suspensions of GO and TrGO were obtained using a UV‐visible spectrophotometer (Shimadzu, Japan). XRD analyses were carried out using an X‐ray diffractometer (Rigaku, Japan). FTIR spectra were recorded in the wavelength range of 4000–400 cm^−1^ using KBr pellets (FTIR SHIMADZU). Zeta potential and particle size distribution were determined using DLS (Horiba Scientific SZ‐100). Structural characteristics were examined using TEM coupled with energy‐dispersive X‐ray spectroscopy analysis (TEM FEI‐TECNAI G2‐20 TWIN).

### 2.6 Biological application

#### 2.6.1 Cell line culture

The MCF‐10A cell line and MCF cell line were maintained in RPMI‐1640 medium supplemented with 10% FBS, 2 mM glutamine, penicillin (100 µL/ml), and streptomycin (100 µL/ml) and incubated at 37°C in a CO_2_ incubator.

#### 2.6.2 *In‐vitro* cytotoxic assay

The cytotoxic effects of GO and TrGO against MCF‐10A and MCF‐7 cell lines were evaluated by MTT assay [31]. The cells (1 x 10^5^ cells/well) were seeded in three sets of 96‐well microtiter plates with 100 μL serum‐free growth medium incubated at 37°C in a CO_2_ incubator for 24 h. After that, the medium was removed, fresh medium containing different concentrations of test compound (3.9 µg/ml to 500 µg/ml) was added and incubated at 37°C in a CO_2_ incubator for 2 days. Negative and positive control were prepared and incubated. The final volume was 100 μL per well. Then MTT reagent (0.45 mg/ml) was added to each well, and the plates were incubated at 37°C in a CO_2_ incubator for 2 h. Once the incubation was completed, the MTT reagent was removed from each well, and dimethyl sulphoxide (DMSO; 75 μL) was added to each well and mixed thoroughly by pipetting. The absorbance was recorded at 540 nm using a plate reader, and the percentage of cell viability was calculated using the formula, % cell viability = A_540_ of treated cells x 100% A_540_ of control cells.

#### 2.6.3 Statistical analysis

All data were presented as mean ± standard deviation (n = 3). One‐way ANOVA was used to analyze the comparison of cytotoxic effect between groups. All statistical analyses were performed using a 95% confidence interval (P < 0.05).

## 3 RESULTS AND DISCUSSION

### 3.1 Spectroscopic characterization

#### 3.1.1 UV‐visible spectra analysis

**FIGURE 1:**
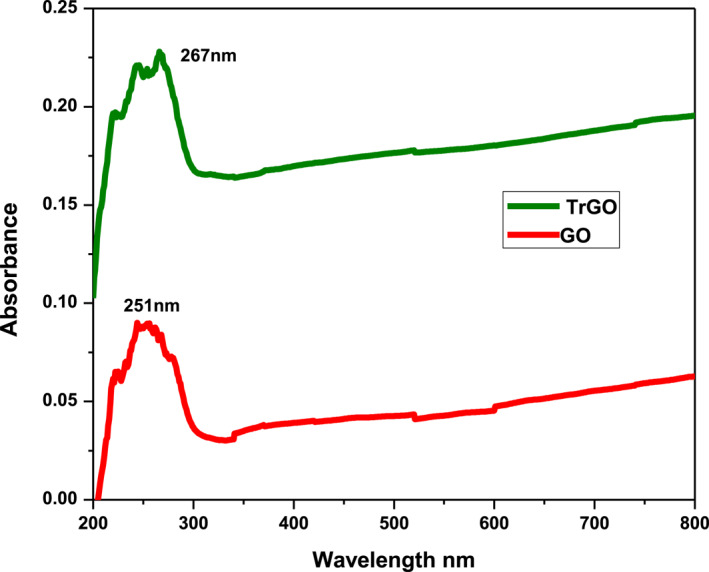
UV‐Visible absorption spectra of GO and T‐rGO. GO, graphene oxide

UV‐visible spectral analysis of GO suspension showed a maximum absorption peak (λ_max_) at 251 nm attributed to π‐π^*^ transitions of the aromatic C=C bond [32]. The transformation of yellow‐brown GO suspension into black colloidal solution indicated successful deoxygenation of the GO suspension under the bioreduction process to form TrGO (Figure 1). This could probably result from an increase in the hydrophobicity of the material caused by a decrease in polar functionality on the surface of the nanosheets [33]. The bioreduced GO (TrGO) exhibited a broad shoulder peak at 267 nm that could be due to n‐π^*^ transition of C=O bonds [34]. This shift in the absorption maxima from 251 to 267 nm points towards the restoration of the sp2‐hybridized carbon network is due to the removal of oxygen‐bearing functional groups [35]. The reduction of GO to graphene increased the π‐conjugation [36]. With the increase in the π‐conjugation, less energy is required for the transition corresponding to the shift in absorption to the longer wavelength region [37]. The effective bioreduction of GO could be attributed to various phytochemicals such as alkaloids, phenols, flavonoids, proteins, lipids, carbohydrates, and glycosides present in the methanol extract of *T. ornata* capped over the surface of reduced GO nanosheets to prevent agglomeration [38]. In the reaction mixture, the biomolecules of *T. ornata* undergo a free radical mechanism by donating hydrogen radical for the successful reduction of GO to rGO [2].

#### 3.1.2 X‐ray diffraction

**FIGURE 2:**
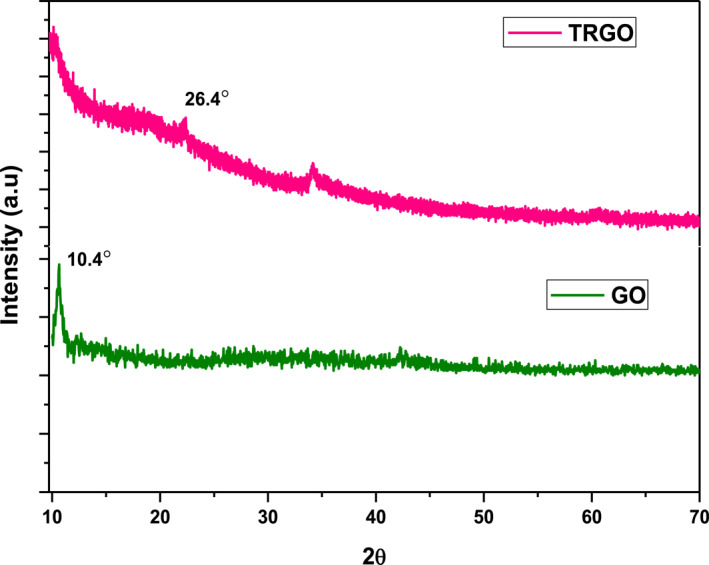
X‐ray diffraction patterns of GO and T‐rGO. GO, graphene oxide

The XRD diffractogram of pristine GO showed a sharp peak at *2θ*‐10.7° corresponding to a Bragg's reflection of (100), which indicated oxidation of graphite from the intercalation of water molecules and establishment of different species of oxygen such as carboxyl, hydroxyl, and epoxy between the graphite sheets [39]. After bioreduction of GO by *T. ornata,* the diffraction peak shifted to *2θ*‐26.4°, corresponding to a Bragg's reflection of (001), which was due to restacking of graphene sheets [40] (Figure 2). The characteristic broad peak was due to the stacking of single or limited layers of amorphous structure of rGO after the reduction. The obtained diffraction spectra were in accord with GO nanosheets that were bioreduced with leaf extracts of *Colocasia esculenta*, *Mesua ferrea Linn,* and *Eucalyptus* [41, 42]. Most interestingly, the disappearance of the peak at 10.7° indicated efficient removal of oxygen‐containing groups in GO after reduction [43].

#### 3.1.3 Fourier transform infrared spectroscopy

**FIGURE 3:**
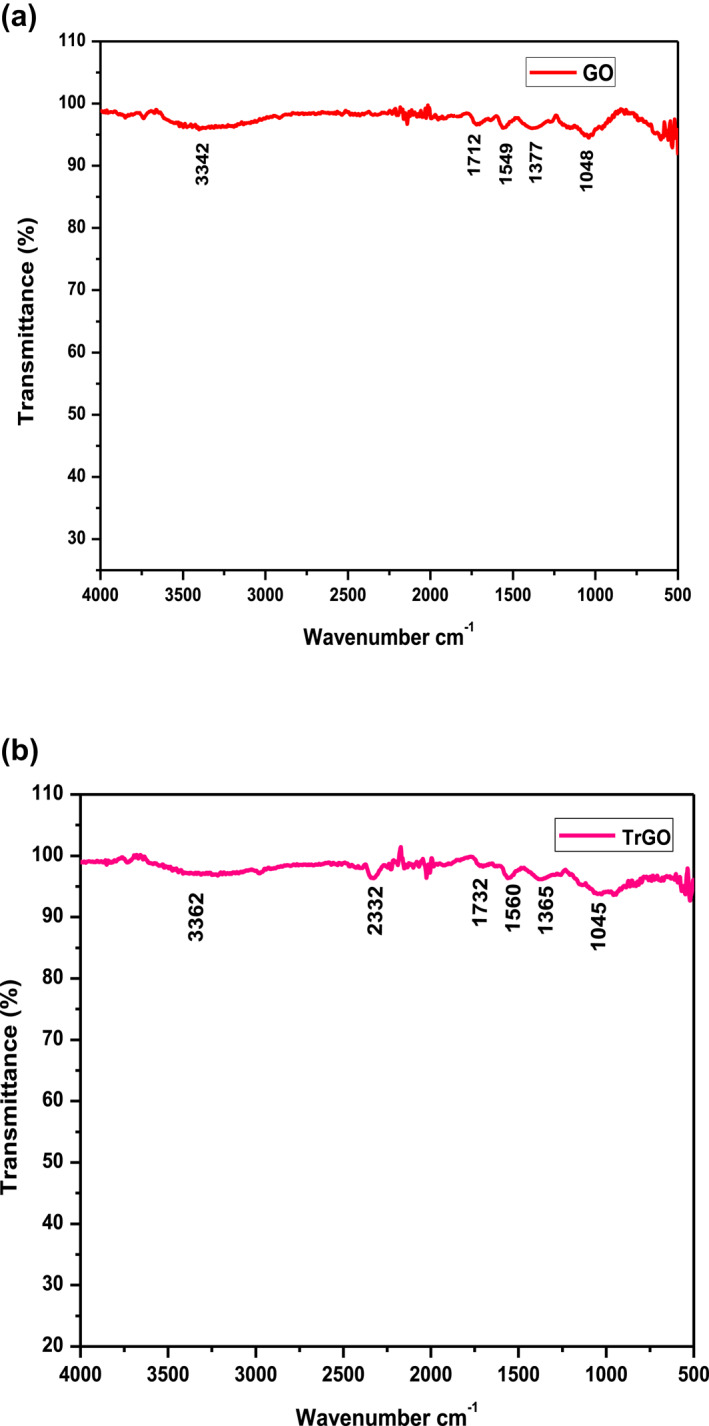
Fourier transform infrared spectra of (a) GO and (b) T‐rGO. GO, graphene oxide

The FTIR spectra of GO and TrGO samples were recorded to identify the extent of reduction (Figure 3a and b). The presence of different oxygen functional groups recorded in the spectra confirmed the oxidation of graphite to GO. Figure 3(a) represents the FTIR spectra of GO. A broad peak at 3342 cm^−1^ corresponds to OH stretching vibrations [2]. A peak located at 1712 cm^−1^ was attributed to C=O (carbonyl/carboxyl functional group) stretching [44]. The absorption peak at 1549 cm^−1^ denoted C=C stretching vibrations [45]. In addition, the spectrum of GO exhibited a peak at 1377 cm^−1^ related to sp^3^ C‐H stretching of saturated carbons [46]. A broad peak at 1048 cm^−1^ signifies C‐O (alkoxy) stretching vibration, respectively [47]. The FTIR spectra of the TrGO (Figure 3b) showed the emergence of an additional absorption peak at 2322 cm^−1^ corresponding to C=C vibrations of aromatic groups [48]. This denotes the effective functionalisation of graphene layers by capping various biomolecules such as phenolic compounds, flavonoids, tannins, and coumarins present in the methanol extract of *T. ornata* [49]. The IR spectral pattern obtained in our study agreed with GO bioreduced by *Aloe vera* extract [50]. With the above IR data, a significant decrease in the absorption band intensities of functional oxygen moieties (OH, C=O, C‐O) was evident, indicating deoxygenation and successful reduction of GO.

#### 3.1.4 Zeta potential

**FIGURE 4:**
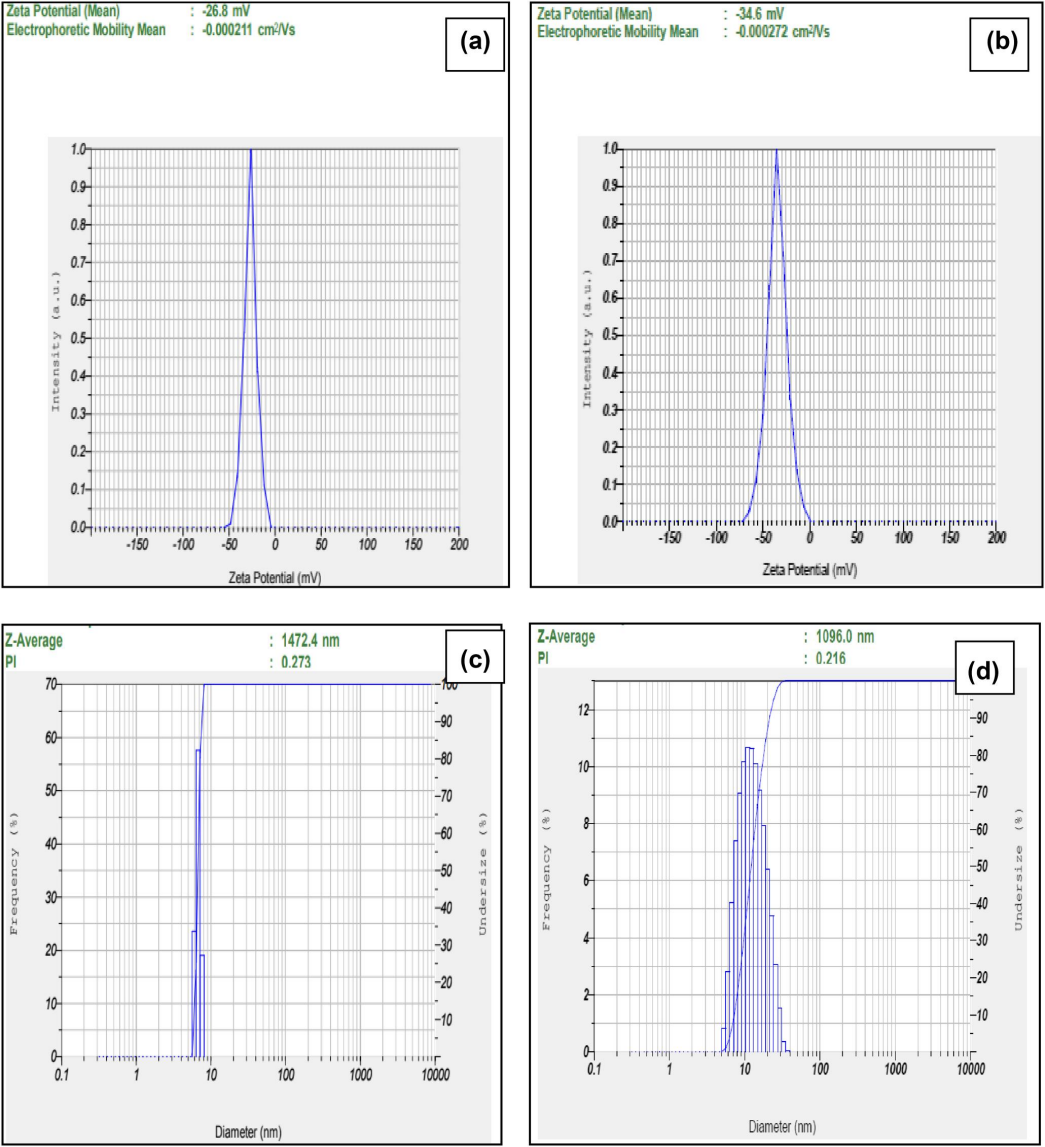
Zeta potential of GO (a); TrGO (b) and hydrodynamic size distribution of (c) GO and (d) T‐rGO measured with DLS spectroscopy. GO, graphene oxide

DLS analysis showed that the zeta potential (ζ) of GO in an aqueous medium was measured to be −26.8 mV, which indicates the presence of a large number of carboxyl groups with highly negative charge density formed in the surface of GO nanosheets [51] (Figure 4a). However, the value has decreased to −34.6 mV after the bioreduction process by *T. ornata* (Figure 4b). The observed pattern of zeta potential (ζ) was in accordance with the bioreduction of GO assisted by heparin [48]. The zeta potential (ζ) value conferred that TrGO particles possess good stability in the aqueous solution. In addition, it is also stated that the zeta potential (ζ) of reduced GO dispersion is pH dependent and is lower than GO [43]. The change in zeta potential (ζ) values indicated that TrGO are functionalized with more surface negative charge than GO. Meanwhile, the average hydrodynamic diameter of GO was measured to be 1472.4 nm, which was decreased to 1096 nm after the bioreduction process (Figure 4c and d). The variation in the surface charge (ζ) and average particle size indicated that *T. ornata* acted as an efficient reducing agent to functionalize the surface of TrGO particles leading to decreased Brownian motion rate after the reduction process [52].

#### 3.1.5 Energy‐dispersive X‐ray spectroscopy

**FIGURE 5:**
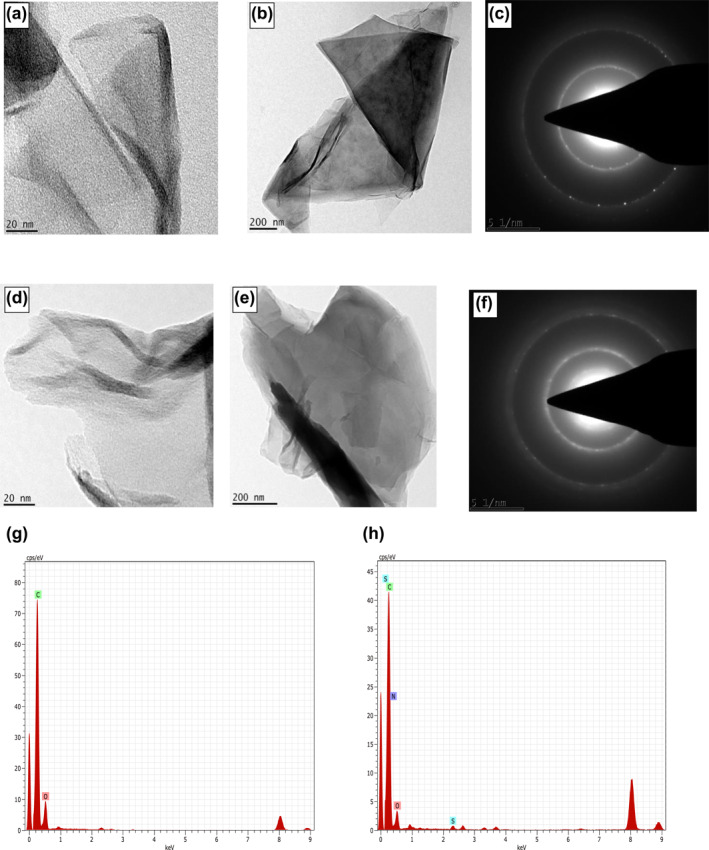
Transmission electron microscopy microscopic images and SAED of GO (a,b,c); TrGO (d,e,f); EDX spectra of (g) GO and (h) TrGO. GO, graphene oxide

The EDX spectra recorded in spot‐profile mode showed intense spectra of carbon atoms in the nanosheets (Figure 5g and h). The absorption spectra of carbon and oxygen in the range of 0 to 1 keV confirmed the presence of TrGO nanocrystallites [53]. The atomic percentages of carbon (C) and oxygen (O) in GO was measured to be 98.60% and 1.40%, respectively. The EDX spectra recorded for TrGO showed C of 98.42% and O of 0.88%, respectively. Comparative analysis revealed that the intensity of the oxygen signal was decreased in TrGO, confirming the partial removal of oxygen‐containing functional groups after the reduction [54].

### 3.2 Morphological analysis

#### 3.2.1 Transmission electron microscopy

The microscopic view of GO and TrGO at lower (20 nm) and higher magnification (200 nm) observed under TEM is represented in Figure 5. Lower magnification of GO at 20 nm revealed a thin layer of GNs appearing as silky veil waves with densely folded scrollings on the edges (Figure 5a), confirming its stability under a high‐energy electron beam [47]. The observed structural features are attributed to the intrinsic nature of graphene [43]. In contrast, TrGO samples appeared as an ultra‐transparent monolayer at 20 nm (Figure 5d). Higher magnification of GO at 200 nm depicted a large triangular‐shaped folded sheet in a thick scrolling pattern along with a fine view of microwrinkling and sharp edges (Figure 5b). In turn, TrGO visualized at 200 nm displayed a clear view of multiple corrugations (Figure 5e). The existence of scrolling and corrugations on the edges of nanosheets could arise from the thermodynamic stability of the two‐dimensional membrane resulting from microscopic crumpling via bending or buckling [33, 55]. The folding pattern observed in both samples could be due to interlayer partial chemical and hydrogen bonds [43].

The morphological variations observed in TEM especially differentiated the dense folded stacking layers of GO and transparent sheet of TrGO with corrugations, which aligned with the variations in the structural features of GO and reduced GO assisted by baker's yeast [56]. The observations further confirm that the biological reduction mediated by *T. ornata* has played a significant role in the transformation of GO to reduced GNs.

The selected area electron diffraction pattern of GO and TrGO shows a typical sharp polycrystalline ring pattern consisting of many diffraction spots that correspond to (111) plane confirming high crystallinity (Figure 5c and f). This result confirms the destruction of interlayer coherence and random orientation of oxygen‐free sheets [39].

### 3.3 *In‐vitro* biological applications

#### 3.3.1 *In‐vitro* antiproliferative assay

**FIGURE 6:**
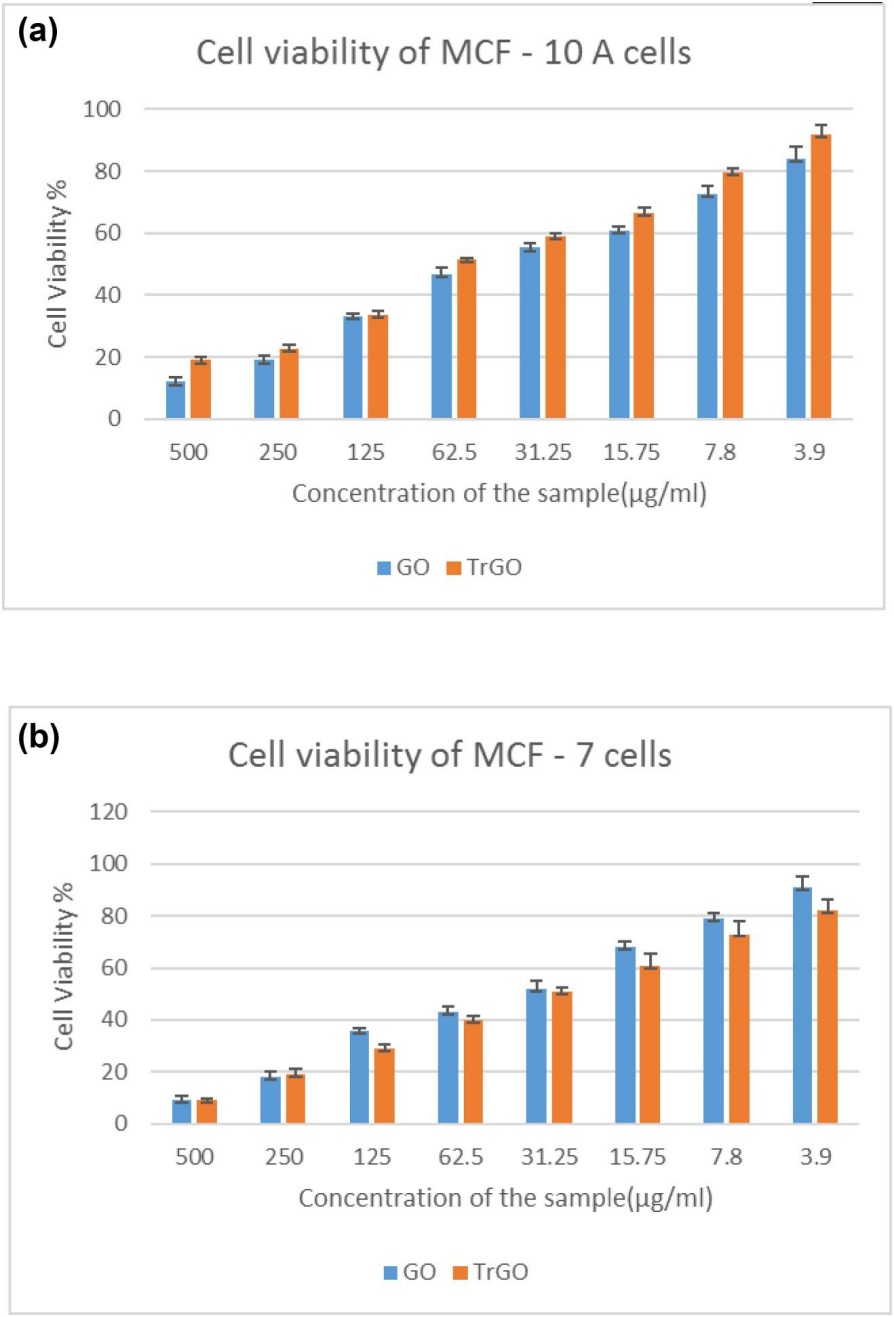
*In‐vitro* cytotoxic effect of (a) GO and (b) T‐rGO against MCF 10 A and MCF‐7 cell lines determined by MTT assay. Data are presented as mean ± SD (n = 3). GO, graphene oxide

The cytotoxic effect evaluated by MTT assay against MCF‐10 A (normal human breast epithelial cell line) and MCF‐7 cell lines (human breast carcinoma) showed concentration‐dependent effects for both treatments (Figure 6a and b), with all data presented as mean ± SD (*n* = 3). GO tested at a minimal concentration of 3.9 µg/ml showed 84% and 92% cell viability of MCF‐10A and MCF‐7 cell lines. Meanwhile, at a maximum concentration of GO (500 µg/ml), the percentage of cell viability was drastically reduced to 10% and 11% and showed no statistically significant difference in cell viability between MCF‐10A and MCF‐7 cells. In contrast, the cell viability of MCF‐10A and MCF‐7 cell lines was recorded to be 91% and 82% in TrGO tested at 3.9 µg/ml. At a high concentration of TrGO (500 µg/ml), the cell viabilities (%) of MCF‐10A and MCF‐7 cell lines were found to decrease to 19% and 9%, respectively, thus indicating a statistically significant difference in cell viability between MCF‐10A and MCF‐7 cells. Interestingly, it was observed that TrGO exerted less cytotoxic effect on MCF‐10A cell lines, which was comparably higher in GO treatment. In addition, it was evident that when compared with MCF‐10A cells, the cell viability (%) of MCF‐7 cell lines has declined in TrGO, and statistically, a significant difference in cell viability between MCF‐10A and MCF‐7 cells was observed in TrGO. Various studies on the application of GO for its anticancer effect against MCF‐7 cell lines such as GO polyethylene glycol‐folic acid conjugated with camptothecin (GO‐PEG‐FA‐CPT) tested at a higher concentration (100 μg/ml) demonstrated 79.92% cell viability [57], An Ag‐GO composite reduced with walnut green husk extract showed cell viability closer to 70% at 60 μg/ml [58]. In concurrence with the previous reports and based on our study results, we observed 29% cell viability at 125 μg/ml and 40% viability at 62.5 μg/ml. In the present study, the IC_50_ of TrGO treatment against MCF‐7 was calculated to be 31.25 µg, which is onefold lower than the cytotoxic effect of methanolic extract of *T. ornata* (60.0 ± 1.14 µg/ml) [59]. With these findings, it became apparent that TrGO has an effective cytotoxic property against MCF‐7.

The cytotoxic effect demonstrated by TrGO against MCF‐7 cell lines could be due to the presence of a good number of phenols and flavonoids in the methanolic extract of *T. ornata* [60]. More specifically, capping of phenolic compounds such as fucoxanthin and fucoidan from *T. ornata* on the surface of nanosheets would enhance the biofunctional effects of TrGO [61]. In addition, the presence of bioactive molecules such as fucan‐like polysaccharide [62] and pseudopelletierine [63] would contribute to the antiproliferative effects of TrGO.

The cell viability of MCF‐7 cells was further confirmed by phase‐contrast microscope (Leica microscope) (photographs are not included). The remaining untreated cells exhibited epithelial‐like morphology with long arms and inconclusive cell boundaries. The TrGO‐treated cells were irregularly shaped and had contracted arms with shrinkage, indicating the progression of apoptosis [64]. The cellular toxicity could be induced by the interaction of TrGO with the plasma membrane or extracellular matrix through diffusion, endocytosis, or binding to receptors [65, 66, 67]. During the interaction with cells, GO facilitates reactive oxygen species leading to oxidative stress, loss of cellular function, proinflammatory responses, and mitochondrial damage [2] The uptake of graphene into the nucleus leads to breakage of DNA strands and induction of gene expression via the activation of transcription factor, ultimately leading to cell death [68]. The observed results firmly support the biocompatible nature of TrGO with MCF‐7 cells for inducing the death of cancerous cells.

## 4 CONCLUSION

A biological synthesis of TrGO nanosheets using methanol extract of marine brown macroalgae *T. ornata* has been discussed. The synthesized TrGO was characterized spectroscopically using different techniques such as UV‐visible; XRD, FTIR, DLS, and EDX. Most importantly, FTIR and EDX spectroscopic techniques clearly depicted the changes in the vibration intensities of oxygen functional moieties and concentration of oxygen (atomic %) in both GO and rGO, confirming deoxygenation as a result of the bioreduction process. TEM displayed variations in the structural characteristics such as presence of microwrinkling and sharp edges in GO and a transparent monolayer with multiple corrugations in TrGO. The zeta potential value of synthesized TrGO conferred that TrGO particles possess good stability. The cytotoxic effects of TrGO on MCF‐7 cell lines was assessed using MTT assay, thus proving that TrGO exerts a cytotoxic effect on breast cancer cell lines.
